# IRES-Mediated Translation of Utrophin A Is Enhanced by Glucocorticoid Treatment in Skeletal Muscle Cells

**DOI:** 10.1371/journal.pone.0002309

**Published:** 2008-06-11

**Authors:** Pedro Miura, Meghan Andrews, Martin Holcik, Bernard J. Jasmin

**Affiliations:** 1 Department of Cellular and Molecular Medicine, Centre for Neuromuscular Disease, University of Ottawa, Ottawa, Ontario, Canada; 2 Apoptosis Research Centre, Children's Hospital of Eastern Ontario, Ottawa, Ontario, Canada; 3 Ottawa Health Research Institute, Molecular Medicine Program, Ottawa Hospital, Ottawa, Onatario, Canada; Universität Heidelberg, Germany

## Abstract

Glucocorticoids are currently the only drug treatment recognized to benefit Duchenne muscular dystrophy (DMD) patients. The nature of the mechanisms underlying the beneficial effects remains incompletely understood but may involve an increase in the expression of utrophin. Here, we show that treatment of myotubes with 6α−methylprednisolone-21 sodium succinate (PDN) results in enhanced expression of utrophin A without concomitant increases in mRNA levels thereby suggesting that translational regulation contributes to the increase. In agreement with this, we show that PDN treatment of cells transfected with monocistronic reporter constructs harbouring the utrophin A 5′UTR, causes an increase in reporter protein expression while leaving levels of reporter mRNAs unchanged. Using bicistronic reporter assays, we further demonstrate that PDN enhances activity of an Internal Ribosome Entry Site (IRES) located within the utrophin A 5′UTR. Analysis of polysomes demonstrate that PDN causes an overall reduction in polysome-associated mRNAs indicating that global translation rates are depressed under these conditions. Importantly, PDN causes an increase in the polysome association of endogenous utrophin A mRNAs and reporter mRNAs harbouring the utrophin A 5′UTR. Additional experiments identified a distinct region within the utrophin A 5′UTR that contains the inducible IRES activity. Together, these studies demonstrate that a translational regulatory mechanism involving increased IRES activation mediates, at least partially, the enhanced expression of utrophin A in muscle cells treated with glucocorticoids. Targeting the utrophin A IRES may thus offer an important and novel therapeutic avenue for developing drugs appropriate for DMD patients.

## Introduction

Glucocorticoid administration is currently the only drug treatment known to offer real clinical benefit to patients suffering from Duchenne muscular dystrophy (DMD). Glucocorticoids used to treat DMD include prednisone [1] and its oxazoline derivative, deflazacort [2]. DMD patients treated with glucocorticoids exhibit delayed progression of muscle weakness [3] and remain ambulatory for a greater period of their lives [4]. The mechanism by which patients benefit from glucocorticoid treatment is not fully understood, although it is thought that the clinical benefits arise in part from the anti-inflammatory and immunosuppressive effects of these drugs [5]. Previous work has shown that deflazacort treatment of the mdx mouse, a dystrophin deficient model of DMD, can alleviate symptoms of the dystrophic pathology and results in the stimulation of utrophin A expression in skeletal muscle fibers [6]. This observation is important since one therapeutic strategy for the treatment of DMD involves the stimulation of endogenous utrophin levels in dystrophic skeletal muscle fibers [7], [8]. In this context, utrophin upregulation represents an interesting therapeutic strategy for DMD since it is the autosomal homologue of dystrophin, the protein missing from DMD muscle fibers. Several previous studies have in fact shown the ability of utrophin to functionally compensate for the absence of dystrophin in various animal models of DMD [9]–[11]. Since stimulation of utrophin expression may be one mechanism by which DMD patients benefit from glucocorticoid treatment, it thus becomes important to define the molecular targets through which these drugs act to increase utrophin expression in muscle cells.

While utrophin expression can be enhanced at the transcriptional level in response to deflazacort treatment in mdx mice [6], several groups have also demonstrated that utrophin is regulated at the translational or post-translational level in response to glucocorticoid treatment. Indeed, it has been shown that treatment of cultured muscle cells with glucocorticoids causes an increase in utrophin protein expression without corresponding changes in utrophin transcript levels [12], [13]. This discrepancy between utrophin protein and mRNA levels occurs also under other conditions, as we first demonstrated in muscle fibers of DMD patients and regenerating mouse muscle fibers [14]. Similar observations have been made when examining utrophin expression in mdx skeletal muscle [15]. Thus, utrophin appears to be regulated by translational and/or post-translational mechanisms under diverse conditions.

Recently, we provided further evidence that translational control can account for the large increase in utrophin protein expression seen during regeneration of mouse skeletal muscle in the absence of concomitant changes in utrophin transcript levels [16]. By using direct injection of monocistronic and bicistronic reporter vectors harbouring the utrophin A 5′ untranslated region (5′UTR) into mouse skeletal muscle, we showed that the utrophin A 5′UTR contains an Internal Ribosome Entry Site (IRES) that is quiescent in adult muscle fibers, but becomes preferentially activated upon the stress of muscle regeneration [16]. IRES-mediated translation is an alternative mechanism of translation initiation that is believed to occur independently of the methyl^7^ guanosine cap structure at the 5′end of an mRNA. This cap-independent mechanism of translation initiation regulates translation of specific eukaryotic mRNAs in response to stressful conditions in cells where cap-dependent translation is compromised [17]. Since glucocorticoid treatment of muscle cells results in an increase in utrophin expression without alterations in mRNA levels, we hypothesized that IRES-mediated translational control is in fact the mechanism responsible for enhanced utrophin expression under these conditions. Here, we tested this hypothesis and demonstrate that PDN treatment enhances the activity of the utrophin IRES that in turn allows for efficient recruitment of utrophin mRNA into polysomes despite the overall inhibition of protein synthesis that is caused by treatment with PDN.

## Results

### Glucocorticoid Treatment of C2C12 Myotubes Stimulates Utrophin Protein Expression Without Affecting mRNA Levels

**Figure 1:**
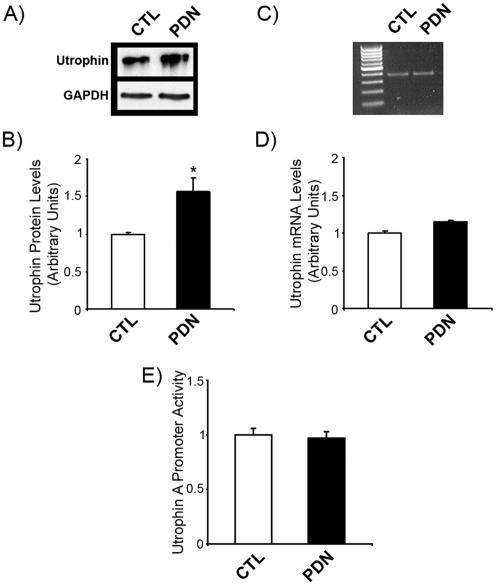
Discordant expression of utrophin and its mRNA in glucocorticoid-treated C2C12 myotubes. C2C12 myotubes were treated with either DMSO (CTL) or 6α-methylprednisolone-21 sodium succinate (PDN) at 500 nM for 3 days. Cells were harvested and protein or RNA was isolated for analysis. A) Western blot analysis for utrophin (drp2 antibody) and GAPDH. B) Quantification of the signal intensity seen in the Western blot experiments shows that utrophin levels are stimulated ∼1.5 fold by PDN treatment. C) Representative ethidium bromide-stained agarose gels showing utrophin PCR product amplified from CTL- and PDN-treated cells. D) Quantification of the RT-PCR product bands shows that utrophin mRNA levels are unchanged by PDN treatment. Levels of utrophin mRNA are standardized to GAPDH mRNA. Values are in arbitrary units. E) Cells were transfected with a utrophin A promoter-reporter construct and treated as above. Quantification of β-galactosidase activity (standardized to a co-transfected pCAT plasmid) demonstrates that the utrophin A promoter is not activated by PDN treatment. Transcriptional mechanisms thus cannot explain the increased expression of utrophin protein following PDN treatment. Mean +/− S.E. are shown. Experiments were performed in triplicate, n = 3. *, significant differences from control (*p*<0.05).

Earlier studies have demonstrated that utrophin is stimulated at the protein, but not mRNA level in muscle cells treated with various glucocorticoids [12], [13]. We thus treated C2C12 myotubes for 3 days with the glucocorticoid 6α-methylprednisolone-21 sodium succinate (PDN) at a concentration previously shown to increase utrophin protein levels by ∼40% (500 nM) [13]. As expected, Western blot analysis showed that utrophin protein levels were ∼1.5 fold greater (*p*<0.05) in PDN- versus DMSO (control: CTL)- treated myotubes (Figure 1A, B). In contrast to this induction upon PDN treatment, and in accordance with previous findings [13], we observed no increase in utrophin mRNA levels by both end point PCR (Figure 1C, D) and quantitative PCR (Figure 4B) (*p*>0.05). Similarly, utrophin promoter activity was not induced by PDN treatment, as revealed by transient transfection experiments using a utrophin A promoter-reporter construct (Figure 1E).

### The Utrophin A 5′-UTR Regulates Expression of a Reporter Protein in Response to Glucocorticoid Treatment

**Figure 2:**
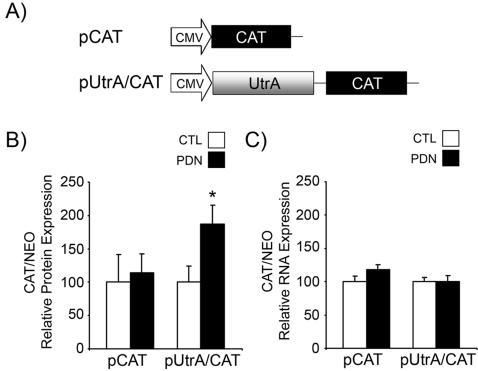
The utrophin A 5′-UTR regulates translation of a reporter protein following PDN treatment of C2C12 myotubes. A) Monocistronic reporter constructs used in these experiments. pCAT is driven by the CMV promoter, pUtrA/CAT contains the utrophin A full-length 5′UTR (UtrA) subcloned upstream of the CAT reporter. C2C12 myoblasts were transfected with either pCAT or pUtrA/CAT constructs, induced to differentiate and then treated for 3 days with PDN (500 nM) or DMSO (CTL). B) Relative CAT protein reporter activity levels. Values are shown as a ratio of CAT reporter protein activity to neomycin (NEO) protein reporter activity. The neomycin reporter is expressed from a separate promoter on the pCAT construct and serves as an internal control. Note the increase in CAT reporter levels in response to PDN treatment when the utrophin 5′UTR is present. C) Relative levels of CAT containing mRNA reporter transcripts, standardized to NEO reporter mRNA levels, as assessed by RT-PCR. Note that in contrast with was is seen with reporter protein levels, CAT reporter RNA levels are unchanged by PDN treatment independent of the presence of the utrophin A 5′UTR. Values are in arbitrary units. Mean +/− S.E. are shown representing the average of three experiments performed in triplicate. *, significant difference from DMSO control (CTL) (*p*<0.05).

We next performed a complementary set of experiments to test the hypothesis that PDN treatment enhances translation of utrophin A transcripts via events targeting its 5′UTR. To this end, we transfected myoblasts with a monocistronic construct driven by the CMV promoter that contained the utrophin A 5′UTR upstream of a CAT reporter (pUtrA/CAT) (Figure 2A). After inducing myoblasts to differentiate, we treated these cells with DMSO (CTL) or PDN for 2–3 days. Levels of CAT reporter protein were then measured and standardized to levels of neomycin protein, expressed from the same plasmid via a separate promoter. The results show that PDN treatment caused an increase in the relative expression of CAT protein by ∼1.5–2 fold (*p*<0.05) in cells transfected with the pUtrA/CAT plasmid. In contrast, CAT protein levels remained unaffected by PDN treatment in cells transfected with the parental pCAT construct (Figure 2B). Quantitative analysis of the levels of CAT and neomycin mRNAs by RT-PCR revealed that relative CAT mRNA levels were not affected by PDN treatment in muscle cells transfected with either the pCAT or pUtrA/CAT construct (Figure 2C). Thus, the observed increase in CAT protein expression without concomitant induction of CAT mRNA expression, strongly suggests that PDN treatment causes increased translation of the UtrA/CAT mRNA.

### Glucocorticoid Treatment Enhances Utrophin A IRES Activity

**Figure 3:**
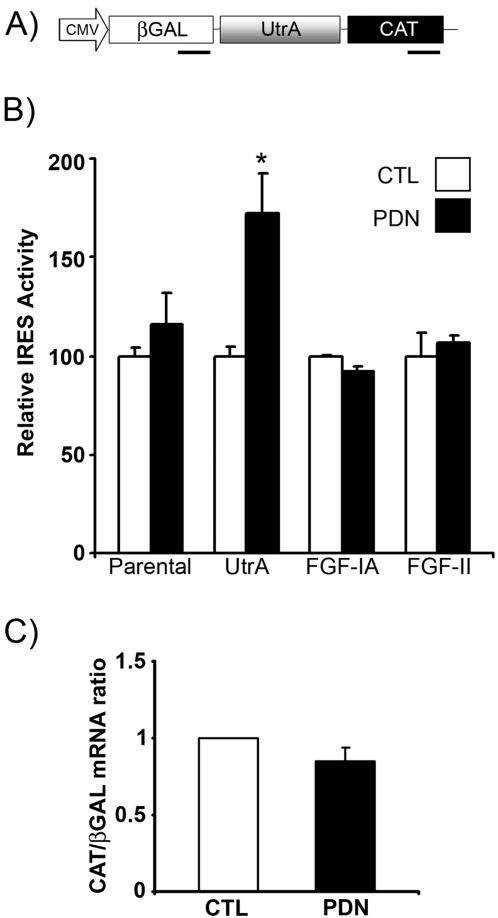
Utrophin A IRES activity is stimulated by PDN treatment of C2C12 myotubes. A) Schematic representation of bicistronic vector containing the utrophin A 5′UTR (pβGAL/UtrA/CAT). Regions amplified by quantitative RT-PCR in control experiments (data presented in C) are indicated with black bars beneath the βGAL and CAT cistrons. B) C2C12 myoblasts were transfected with either an empty vector bicistronic reporter construct (parental), or bicistronic reporter constructs harbouring IRES elements from FGF-IA, FGF-II and utrophin A (UtrA). Cells were induced to differentiate and then treated for 3 days with PDN or DMSO (CTL). Relative IRES activity is reported as a ratio of CAT (measuring cap-independent translation), to βGAL (measuring cap-dependent translation). Data from PDN-treated samples are standardized to control, DMSO-treated samples. See Table 1 for individual expression levels of the βGAL and CAT cistrons. Mean +/− S.E. are shown representing the average of four experiments performed in triplicate. *, significant difference from DMSO control (CTL) (*p*<0.05). Note that the effect of PDN is specific for the utrophin A 5′UTR. C) Quantitative RT-PCR using βGAL and CAT primers was performed on RNA from treated C2C12 cells transfected with pβGAL/UtrA/CAT.to exclude the possibility of spurious splicing or cryptic promoter activity of the utrophin A IRES. The lack of significant difference in the ratio of CAT to βGAL cDNA products between CTL- and PDN-treated cells indicates, as expected, the presence of an intact bicistronic transcript. The CAT/βGAL ratio is expressed as 2^−[Ct(CAT)−Ct(βGAL)]^ (see materials and methods for details). Values are in arbitrary units. Mean +/− S.D. are shown representing the average of three experiments performed in triplicate.

In subsequent experiments, we determined whether enhanced translation mediated by the utrophin A 5′UTR in response to PDN treatment results from induced IRES activity. For these studies, we used bicistronic reporter constructs and assays as described previously [16]. C2C12 myoblasts were first transfected with either a bicistronic construct containing the utrophin A 5′UTR inserted between the two cistrons (pβGAL/UtrA/CAT) (Figure 3A) or an empty vector control (pβGAL/CAT). Increased expression of the downstream cistron (CAT), relative to the upstream cistron (βGAL) provides an indication of enhanced IRES activity. Assessment of utrophin A IRES activity in cells treated with PDN at concentrations ranging from 5 nM to 1 mM revealed that maximal utrophin A IRES activity was induced at 500 nM (data not shown). As shown in Figure 3B, 500 nM PDN treatment of muscle cells transfected with pβGAL/UtrA/CAT led to a significant ∼1.5 fold increase (*p*<0.05) in IRES activity as measured by an increased ratio of CAT to βGAL. This increase in relative IRES activity of the pβGAL/UtrA/CAT transfected cells is due to a stimulation in expression of the downstream cistron thus reflecting a genuine increase in IRES activity (Table 1). This level of induction in the activity of the utrophin A IRES is consistent with the observed increase in endogenous utrophin protein levels following treatment with PDN (See Figure 1A, 1B and [13], [18]). In contrast to the enhancement of utrophin IRES activity, the activity of the FGF-IA [19] and FGF-II [20] IRES elements were not enhanced by PDN treatment ( *p*>0.05).

**Table 1:** Individual βGAL and CAT expression levels for cells transfected with parental or utrophin A 5′UTR bicistronic constructs and treated with DMSO or PDN.

Construct	Treatment	βGAL	CAT
Parental	DMSO	100.0 +/− 2.0	100.0 +/− 0.8
Parental	PDN	103.7 +/− 5.2	117.4 +/− 15.5
UtrA	DMSO	100.0 +/− 3.9	100.0 +/− 6.2
UtrA	PDN	97.8 +/− 3.0	167.8 +/− 19.1

Values correspond to the relative levels of IRES activity shown in Figure 3B for cells transfected with pβGAL/CAT (Parental) or pβGAL/UtrA/CAT (UtrA). Note the increase of CAT activity when the UtrA transfected cells are treated with PDN. Values are standardized to DMSO treated condition. Mean +/− S.E. is shown.

We previously demonstrated that the utrophin A 5′UTR contains a *bona fide* IRES element, and that the reported IRES activity as measured using bicistronic vectors was not due to the presence of an internal promoter or spurious splicing events [16]. To ensure that the observed induction of utrophin A IRES activity seen here in response to PDN treatment was due to a genuine increase in cap-independent translation, we performed quantitative RT-PCR analysis on cells transfected with pβGAL/UtrA/CAT and treated with PDN or DMSO as control. Analysis of the amplification profiles of the βGAL and CAT cDNA products revealed no change in the proportion of βGAL to CAT cistron RNA in PDN- versus DMSO-treated cells (Figure 3C). In addition, we transfected a utrophin bicistronic construct lacking the CMV promoter, p(-cmv)βGAL/UtrA/CAT [16], and found that CAT activity was not induced by PDN treatment (*p*>0.05) (data not shown). Together, these experiments indicate that the PDN-induced increase in IRES activity is not a result of aberrant splicing or cryptic promoter activity.

### Analysis of Polysome-Bound Utrophin A Transcripts During Glucocorticoid Treatment

**Figure 4:**
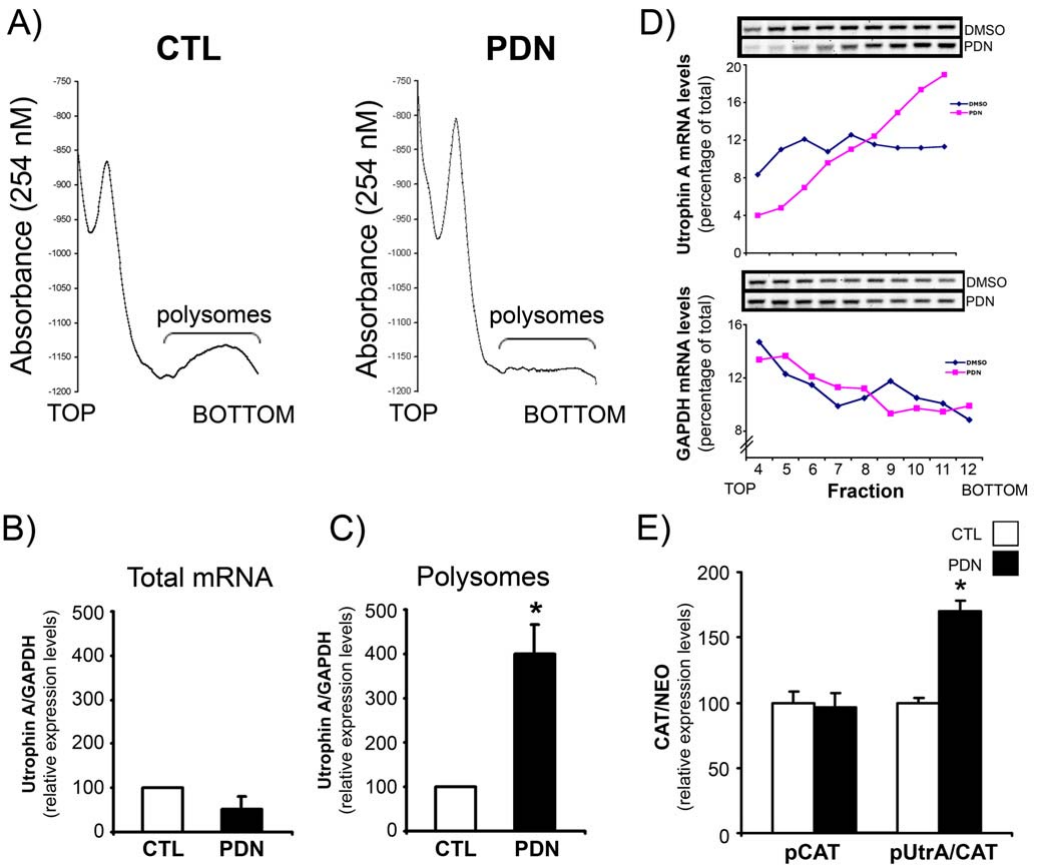
Utrophin A mRNA becomes enriched in polysomes in response to PDN treatment. A) Representative polysome profiles of C2C12 myotube cell lysates fractionated by sucrose density ultracentrifugation. C2C12 myotubes were treated with DMSO (CTL) or with PDN (500 nM) for two days. Cells were then treated with cycloheximide and lysed. Nuclei and cellular debris were removed by centrifugation. Cell lysates were layered on a continuous sucrose gradient. Absorbance of the gradients was measured at 254 nm from top to bottom via a peristaltic pump and RNA-containing sucrose gradients were fractioned for a total of 12 fractions. Note the decrease in polysomal RNA in PDN-treated cells, indicating decreased global protein translation. B) The overall change in endogenous utrophin A mRNA levels in response to PDN-treatment was measured by qRT-PCR from total mRNA samples and is shown relative to GAPDH mRNA, n = 3. C) The change in endogenous utrophin A mRNA levels in the pooled polysome fractions from cells treated with DMSO or PDN relative to GAPDH mRNA. Note the increased presence of utrophin A transcript in the polysome fraction of PDN treated cells, n = 3. D) RT-PCR was performed to detect utrophin A and GAPDH mRNA in individual polysome enriched fractions (fractions 4–12) of the sucrose gradient. Quantification of the amplified bands (See Materials and Methods) demonstrates a shift of utrophin A mRNA to the heavier polysome fractions of the gradient for the PDN treated cells. Data are representative of three independent experiments. Results were confirmed by qPCR. E) Cells were transfected with pCAT and pUtrA/CAT (see Figure 2A) and treated as above. qRT-PCR analysis of CAT reporter transcript expression standardized to neomycin (NEO) reporter transcript expression was carried out on mRNA isolated from the pooled polysome fractions. Note the increase in polysome-associated CAT reporter transcript in response to PDN-treatment, n = 3. All values are in arbitrary units. Mean +/− S.E. are shown. *, significant difference from control (*p*<0.05).

Previous studies have shown that glucocorticoids can cause inactivation of key members of the translational machinery in muscle cells [21] and compromise protein synthesis in skeletal muscle [22]. In order to assess whether PDN treatment of C2C12 myotubes attenuates global translation we fractionated muscle cell lysates by sucrose density gradient centrifugation. In Figure 4A, the optical density profiles of typical sucrose gradients are shown. The top of the gradient contains free mRNAs and ribosomal subunits (40S, 60S, 80S) while the bottom of the gradient contains mRNAs associated with polysomes. Analysis of the optical density profiles demonstrated a reduction in the relative amount of mRNAs associated with polysomal fractions in PDN-treated cells thereby indicating an overall attenuation of protein synthesis under these conditions (Figure 4A).

We next compared the expression profile of utrophin A mRNA in total cellular mRNA extracted prior to sucrose density ultracentrifugation to that of mRNA isolated from the pooled polysome fractions of both DMSO- (CTL) and PDN-treated myotubes. As expected (see Figure 1C and D), quantitative RT-PCR analysis revealed that expression of utrophin A mRNA was not changed by PDN treatment in the total cellular mRNA samples (Figure 4B). In contrast, we observed an ∼4-fold enrichment in the levels of endogenous utrophin A transcripts relative to GAPDH in polysome fractions from PDN-treated myotubes versus controls (Figure 4C). This comparison between total levels of utrophin A transcripts and their association with polysomal fractions indicates that PDN promotes the recruitment of utrophin A mRNA to polysomes despite a general depression in protein synthesis. Such recruitment of utrophin A transcripts to polysomes is further indication that PDN treatment causes a translational induction of utrophin expression. In order to examine the distribution of utrophin A mRNA within the polysome fractions, we performed RT-PCR analysis on individual ∼0.5 mL fractions from the polysome enriched regions of gradients obtained from control and PDN-treated cells (fractions 4–12). As shown in Figure 4D, we found that utrophin A mRNA shifted toward the heavier polysome fractions in PDN treated cells. In contrast, the distribution of GAPDH mRNAs in polysomes were unaffected by PDN treatment (Figure 4D) confirming the observation that GAPDH protein levels remain unaffected in cells treated with PDN (Figure 1A). This shift in distribution to the heavier polysome fractions is further indication that utrophin A translation is enhanced by glucocorticoid treatment.

### The Utrophin A 5′UTR is Responsible for Recruitment of Utrophin A Transcript to Polysomes Upon Glucocorticoid Treatment

Based on these findings, we decided to examine whether the utrophin A 5′UTR could promote the recruitment of reporter transcripts to polysomes upon PDN treatment. We thus transfected myoblasts with the pUtrA/CAT or pCAT monocistronic constructs (Figure 2A), differentiated cells into myotubes and then treated them with DMSO or PDN. Polysome fractionation experiments were performed as described above, and relative levels of CAT- and neomycin-containing mRNA were quantified by RT-PCR. This analysis revealed that PDN treatment caused a significant recruitment (*p*<0.05) of reporter mRNA expressed from the pUtrA/CAT construct to polysomal fractions (Figure 4E). In contrast, no change in the levels of polysome-associated reporter mRNAs was observed following PDN treatment of cells transfected with the parental pCAT plasmid that lacks the 5′UTR (Figure 4E). Thus, the utrophin A 5′UTR plays a key role in recruiting endogenous utrophin A transcripts to polysomes upon PDN treatment.

### Mapping of the Glucocorticoid-Responsive Utrophin A IRES Element

**Figure 5:**
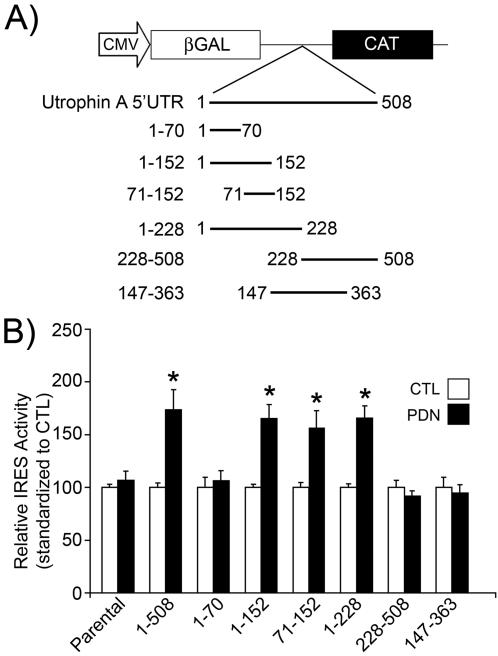
Elucidation of the minimal PDN-responsive utrophin A IRES element. A) Schematic representation of bicistronic constructs harbouring truncated regions of the utrophin A 5′UTR. B) Cells were transfected with the various bicistronic constructs, treated with DMSO (CTL) or PDN (500 nM) for 2 days and analyzed for IRES activity, reported as a ratio of CAT to βGAL protein reporter activity. IRES activity in PDN treated cells is standardized to IRES activity in CTL cells. See Table 2 for individual expression levels of the βGAL and CAT cistrons for each construct and treatment. Mean +/− S.E. are shown representing the average of 3–5 independent experiments, performed in triplicate. *, significant difference from control (*p*<0.05).

In order to delineate the region of the utrophin A 5′UTR that confers PDN-induced IRES activity, several truncated variants of the utrophin A 5′UTR were created and inserted into the bicistronic vector (Figure 5A). Interestingly, PDN treatment was found to enhance IRES activity in muscle cells transfected with bicistronic constructs containing the first 152 or 228 nucleotides of the utrophin A 5′UTR by ∼1.5 fold *p*<0.05) (Figure 5B). This level of induction is similar to that observed using the full-length 5′UTR, i.e., construct containing nucleotides 1–508 in Figure 5B (see also Figure 2B). In contrast, the 147–363 region of the 5′UTR, as well as the 228–508 region did not confer enhanced IRES activity in response to PDN. Further truncation of the 5′UTR from its 3′ end to the first 70 bases (1–70) also abolished the responsiveness to PDN. These experiments suggested that nucleotides 71–152 encompass the PDN-responsive IRES element. Indeed, transient transfection of a bicistronic construct harbouring this region demonstrated an ∼1.5 fold induction in IRES activity upon PDN treatment (*p*<0.05). Individual expression levels of the βGAL and CAT cistrons for each construct and treatment are shown in Table 2. These data demonstrate that the 71–152 fragment can recapitulate the PDN-mediated induction in IRES activity seen with the full-length 5′UTR.

**Table 2:** Individual βGAL and CAT expression levels for cells transfected with bicistronic constructs harbouring truncated regions of the utrophin A 5′UTR and treated with DMSO or PDN.

Construct	Treatment	βGAL	CAT
Parental	DMSO	100.0 +/− 1.9	100.0 +/− 2.9
Parental	PDN	102.0 +/−6.0	104.1 +/− 9.1
1–508	DMSO	100.0 +/− 3.3	100.0 +/− 4.8
1–508	PDN	89.5 +/− 8.6	137.5 +/− 14.3
1–70	DMSO	100.0 +/−6.6	100.0 +/− 6.7
1–70	PDN	94.6 +/− 4.5	102.7 +/− 8.5
1–152	DMSO	100.0 +/− 1.4	100.0 +/− 1.6
1–152	PDN	76.0 +/− 5.7	122.5 +/− 7.2
71–152	DMSO	100.0 +/− 5.0	100.0 +/− 1.6
71–152	PDN	110.2 +/− 8.0	169.0 +/− 9.1
1–228	DMSO	100.0 +/− 2.2	100.0 +/− 3.9
1–228	PDN	75.4 +/− 5.2	120.6 +/− 3.2
228–508	DMSO	100.0 +/− 3.9	100.0 +/− 4.3
228–508	PDN	84.8 +/− 4.4	78.3 +/− 4.4
147–363	DMSO	100.0 +/− 3.8	100.0 +/− 7.2
147–363	PDN	84.1 +/− 4.9	84.8 +/− 10.7

Values correspond to the levels of relative IRES activity for the various constructs shown in Figure 5. Values are standardized to DMSO treated condition. Mean +/− S.E. is shown.

## Discussion

Several groups have previously observed a discordant expression pattern of utrophin protein and mRNA in response to glucocorticoid treatment. Initial studies showed that treatment of cultured muscle cells from DMD patients with the glucocorticoid dexamethasone resulted in increased utrophin protein expression while leaving the level of utrophin transcripts unaffected [12]. Similarly, treatment of normal and dystrophin-deficient myotubes with PDN caused an increase of utrophin protein by approximately 40% in both cell types without altering mRNA levels [13]. Furthermore, various traditional Chinese medicines that tested positive for glucocorticoid-like activity can also stimulate utrophin protein expression in muscle cells [18]. Collectively, these studies suggest that gene regulation at the level of transcription cannot explain the stimulation of utrophin protein expression in response to glucocorticoids.

In the present study, we thus examined whether translational regulatory mechanisms could account for the increased expression of utrophin protein following glucocorticoid treatment. Using multiple and complementary approaches, we show that an enhancement of IRES-mediated translation of utrophin A can account, at least partially, for the increased expression of utrophin A protein in response to PDN treatment of C2C12 myotubes. These results are important particularly in the context of identifying small molecules that could upregulate the endogenous levels of utrophin A in muscle from DMD patients since they show that known drugs can indeed increase the activity of the utrophin A IRES thereby promoting protein synthesis from a pool of already synthesized and translationally competent transcripts. Together with our previous findings obtained in regenerating muscle fibers [16], our current data further illustrate that the utrophin A IRES can be activated by physiologically relevant stimuli.

The enhancement of utrophin translation in response to PDN treatment, as assessed by both monocistronic (Figure 2) and bicistronic (Figure 3) reporter transfection experiments, was found to be similar to the enhancement of endogenous utrophin protein expression in C2C12 cells (Figure 1 A,B). Nonetheless, the increase in utrophin protein levels might also be partially accounted for by an increase in utrophin protein stability. We thus attempted to assess the contribution of protein stability to the enhancement of utrophin expression in response to PDN treatment by treating cells with PDN and cycloheximide. Unfortunately, we found that 48–72 hrs of cycloheximide treatment (10 µg/mL) did not significantly reduce utrophin protein levels as assessed by western blot (see also [23]), thus precluding a thorough assessment of the contribution of protein stability to the enhancement of utrophin expression by PDN treatment.

Several groups have observed that glucocorticoid treatment can cause a reduction in global translation rates. This has in fact been shown to occur in both cultured muscle cells as well as in skeletal muscle of live animals [21], [24]–[26]. The mechanisms by which translation rates are depressed by glucocorticoids in muscle cells have been linked to multiple pathways including inhibition of ribosomal S6 protein kinase (P70 S6K) activity [21], [26] and dephosphorylation of eukaryotic initiation factor 4E binding protein 1 (4E-BP1) [24], [25].

Using sucrose gradient sedimentation, we also observed a reduction in the amount of RNA present within polysomal fractions suggesting a decrease in overall translation. In contrast, both endogenous utrophin A transcripts and reporter mRNAs containing the utrophin A 5′UTR, became enriched within polysomes under these conditions. It is thus interesting to note that utrophin A translation is clearly enhanced by a treatment that simultaneously compromises global translation rates. Several mammalian mRNAs have been shown to exhibit enhanced IRES-mediated translation under conditions where global translation rates are depressed such as during viral infection, mitosis and ER stress [27]–[29]. Our current findings showing an activation of the utrophin A IRES following PDN treatment, fit extremely well with these earlier observations. Together, these results suggest that IRES elements provide an alternative route by which specific mRNAs continue to be translated when the proper functioning of the cap-dependent translational machinery becomes compromised.

While glucocorticoid treatment downregulates global protein synthesis in skeletal muscle cells, several lines of evidence have shown that it can stimulate expression of several proteins at the translational level [30]–[33], including the Na^+^/K^+^-ATPase α1 subunit, which appears to be translationally regulated by glucocorticoids via its 5′UTR [34]–[36] (P.M., M.H., B.J.J., unpublished observations). Along these lines, it is important to note that another steroid hormone, testosterone, has been shown to alter IRES-mediated translation of the Fibroblast Growth Factor 2 (FGF-II) mRNA. In this case, it was found that treatment of an FGF-II IRES reporter mouse with testosterone activated the FGF-II IRES in a manner dependent upon the availability of androgen receptors [37]. Interestingly, testosterone treatment also caused an increase in the binding and interaction of multiple proteins from testes to an FGF-II 5′UTR probe [37].

The nature of the exact mechanisms causing an increase in the activity of the utrophin IRES following PDN treatment of muscle cells remains for the moment unclear. Previous work by others has shown that the increase in utrophin protein expression in response to PDN treatment can be inhibited by simultaneously treating cells with the glucocorticoid receptor antagonist RU486 [13], [18] thereby indicating that this increase is dependent upon activation of the glucocorticoid receptor. It may thus be envisioned that activation of the glucocorticoid receptor stimulates signaling pathways that modulate the association of RNA-binding proteins to the utrophin 5′UTR, thereby enhancing IRES-mediated translation.

A common set of RNA-binding proteins that are required for IRES-mediated translation to occur has not been identified, although several proteins, termed IRES-trans activating factors (ITAFs), have been shown to modulate the activity of different IRESes. These proteins include, for example, polypyrimidine-tract binding protein (PTB) [38], [39], the caspase-cleaved form of DAP5/p97 (p86) [40], [41] and heterogeneous nuclear ribonucleoprotein (hnRNP) A1 [42], [43]. Future studies will aim to identify the ITAFs that mediate IRES-driven translation of utrophin during both glucocorticoid treatment and muscle regeneration.

Treatment of DMD patients with the corticosteroids prednisone and deflazacort slow the progression of the muscle disease. However, this effect is not sustained and the treatment is associated with many detrimental side effects such as weight gain, behavioural abnormalities and osteoporosis [44], [45]. Thus, glucocorticoid treatment does not constitute a viable long-term treatment for DMD. Nonetheless, this treatment increases the length of time a patient remains ambulatory while increasing muscle mass [46] and improving pulmonary function [47], [48]. Therefore, an increased understanding of the mechanisms by which glucocorticoids improve muscle function in DMD patients is critical in ultimately designing similar drugs with longer lasting benefits and fewer side effects.

In addition to utrophin upregulation, it is important to note that glucocorticoid treatment appears to benefit dystrophic muscle via anti-inflammatory actions, including reducing macrophage and T cell (CD2+ and CD8+) accumulation in skeletal muscle [49], [50]. Therefore, the beneficial effect of prolonged glucocorticoid treatment for DMD patients likely stems from the combinatorial impact associated with utrophin upregulation and the anti-inflammatory response.

Given the role of the utrophin A 5′UTR in mediating the translational response to PDN treatment demonstrated here, it may prove useful to begin screening for drugs that target and stimulate utrophin A IRES activity. Based on previous work assessing the therapeutic potential of utrophin to mitigate the dystrophic phenotype of the mdx mouse, such a drug would have to stimulate utrophin expression levels only by ∼2-fold in order to show dramatic improvement in muscle function [51]–[53].

## Materials and Methods

### C2C12 Cell Culture and Drug Treatments

C2C12 myoblasts (American Type Culture Collection, Manassas, VA) were grown as previously described [16] on matrigel-coated plates (BD Biosciences, Bedford, MA) in Dulbecco's modified Eagle's medium (Invitrogen, Carlsbad, CA) supplemented with 10% Fetal Bovine Serum (Cansera, Toronto, ON, Canada) and 100 units/mL of ampicillin/streptomycin. Cells were kept at 37°C in a water-saturated atmosphere containing 5% CO_2_. Upon reaching 80–90% confluency, the growth media was replaced with differentiation media containing 2% Horse Serum (Invitrogen). Cells were maintained in differentiation medium for 2–3 days. For drug treatments, 6α-methylprednisolone-21 sodium succinate (referred to as PDN) (Sigma, St. Louis, MO) was dissolved in DMSO to make 500 µM stocks. DMSO (used as control; CTL) or PDN was mixed with differentiation media at concentration of 500 nM and added to the cells [13]. Fresh media and drug were added each day for 2–3 days of treatment before cell harvesting. In order to monitor the effectiveness of the drug treatments, transient transfections of luciferase reporter constructs harbouring the hormonal response region of the mouse mammary tumor virus promoter [54] were performed.

### Western Blot Analysis

Proteins were isolated from myotubes using a protein lysis buffer and Western blot analysis was carried out using standard procedures as previously described [16]. For detection of protein levels, samples were run on 5% SDS-PAGE gels and transferred onto PVDF membranes. The monoclonal anti-utrophin Drp-2 antibody (Vector Laboratories, Burlingame, CA) was used as at 1∶500 dilution to detect utrophin, followed by incubation with a secondary goat anti-mouse HRP antibody at 1∶2500 (Chemicon International, Temecula, CA). To detect GAPDH protein levels, a monoclonal anti-GAPDH antibody (Advanced Immunochemical, Long Beach, CA) was used at 1∶10000, followed by a secondary goat anti-mouse antibody at 1∶5000 (Jackson Immunoresearch Laboratories, West Grove, PA). Antibody complexes were detected using western lightning chemiluminescent reagent (Perkin Elmer, Waltham, MA) and exposed to Kodak BioMAX MR film (Eastman Kodak, Rochester, NY). The net intensity of the protein bands was quantified using Kodak digital science 1D version 3.0.2 image analysis software.

### Plasmids and Transient Transfections

The bicistronic constructs pβGAL/CAT, pβGAL/UtrA/CAT and p(-cmv)βGAL/UtrA/CAT have been previously described [16]. Progressive deletions of the utrophin A 5′UTR were amplified by PCR and subcloned into the *Xho1* site of pβGAL/CAT. For creation of the monocistronic constructs pCAT and pUtrA/CAT, the LacZ gene was excised from pβGAL/CAT and pβGAL/UtrA/CAT using *Not1* and the vector was re-ligated. The 1.3 kb utrophin A promoter reporter construct used has been previously described [55]. For promoter reporter transfections, a plasmid encoding the CAT gene driven by the SV40 promoter (Promega, Madison, WI) was co-transfected to control for transfection efficiency . The FGF-II IRES was PCR amplified from an FGF-II bicistronic reporter construct (kindly provided by Dr. A.C. Prats, INSERM, Tolouse, France) [19], and subcloned into pβGAL/CAT. A dual luciferase bicistronic construct harbouring the FGF-IA IRES was kindly provided by Dr. A.C. Prats [20]. All transient transfections were carried out using Lipofectamine 2000 (Invitrogen) while the myoblasts were 70–90% confluent as recommended by the manufacturer. For transfections of cells in 35 mm wells, 4 µg of plasmid DNA was combined with 10 µl of Lipofectamine 2000. For experiments involving transfection of monocistronic constructs and analysis of the association of transcripts with polysomes, ten 100 mm plates were transfected for each plasmid and drug treatment combination. Each 100 mm plate was transfected using 24 µg of plasmid DNA combined with 60 µl of Lipofectamine 2000.

### Reporter Protein Analysis

Cells were harvested in Reporter Lysis Buffer (Promega), and assays for β−galactosidase and CAT protein reporter activity were carried out as previously described [16], [56]. For cells transfected with monocistronic constructs, expression of neomycin was detected using the PathoScreen NPTII ELISA assay detection kit as recommended by the manufacturer (Agdia, Elkhart, IN). For transient transfection experiments using the FGF-IA IRES construct, LucR and LucF activities were measured with the dual luciferase kit (Promega).

### Polysome Fractionation Experiments

Polysome fractionation experiments were carried out according to a previously described protocol [57]. C2C12 myotubes were grown on 100 mm plates coated with matrigel and treated with DMSO or PDN as described above. To isolate polysomes, cells were treated with cycloheximide (CHX) (0.1 mg/mL) for 3 minutes in fresh differentiation media. Cells were washed twice in PBS containing CHX and subsequently lysed in an RNA lysis buffer (0.3M NaCl, 15 mM MgCl_2_, 15 mM Tris pH 7.4, 1% Triton X-100, 0.1 mg/mL CHX, 100U/mL RNAsin). Nuclei and cellular debris were removed by centrifugation steps of 3,000 rpm, 5 min, 4°C and then 14,000 rpm, 5 min, 4°C). Three hundred µL of RNA was extracted from part of the lysate for total RNA analysis. Seven hundred µL of the lysate was layered on a continuous sucrose gradient (10–50% sucrose in 15 mM MgCl_2_, 15 mM Tris pH 7.4, 0.3 M NaCl). Centrifugation was carried out at 39,000 rpm in an SW41-Ti rotor at 4°C for 90 min. Absorbance of the gradients was measured continuously at 254 nm from top to bottom of the gradient via a peristaltic pump operating at a flow rate of 1 ml/min. For some experiments, 1 ml fractions were collected and polysome enriched fractions were pooled prior to RNA extraction. To achieve better separation of polysomes, fractions were collected in 0.5 or 1 mL intervals. For fractions 1–3, ∼1 mL was collected per fraction, while for the polysome enriched fractions 4–12, ∼0.5 mL was collected per fraction. The polysome fractions were digested with proteinase K. RNA was extracted by phenol chloroform extraction, and then concentrated and treated with DNAse I using the Absolutely RNA miniprep kit (Stratagene, La Jolla, CA). For RT-PCR analysis (see below) on individual fractions obtained from the sucrose gradient, the signal intensity from cDNA products of each band was divided by the sum of the signal intensities of all bands in order to obtain the percentage intensity of signal in each fraction.

### RNA Extraction and RT-PCR Analysis

Total RNA was isolated from C2C12 cells using TRIzol reagent (Invitrogen) as recommended by the manufacturer. TRIzol extracted RNA was treated for 1 hour with DNAse I (Invitrogen) to eliminate possible plasmid and genomic DNA contamination. To quantify levels of endogenous utrophin and GAPDH mRNAs, RT-PCR analysis was employed using previously described protocols and primer sets [14], [58]. For RT-PCR analysis on cells transfected with the monocistronic pCAT and pUtrA/CAT constructs, CAT was amplified using previously described primers [16] and these levels were standardized to neomycin mRNA levels (5′-TGCTCCTGCCGAGAAAGTAT-3′; 5′-AATATCACGGGTAGCCAACG -3′). Cycle numbers varied depending on the primers used and they were all within the linear range of amplification. Controls consisted of RT mixtures in which total RNA was replaced by sterile, diethylpyrocarbonate-treated water. PCR products were separated on 1.5% agarose gels containing ethidium bromide, and the intensity of the signal, which is linearly related to the amount of cDNAs, was quantified using Kodak digital science 1D version 3.0.2 image analysis software.

In separate experiments, we also performed real-time PCR. For these, analysis of endogenous utrophin A and GAPDH levels was carried out using the following primer sets: utrA (5′-ATCTTGTCGGGCTTTCCAC-3′; 5′- ATCCAAAGGCTTTCCCAGAT-3′); GAPDH (5′-GGGTGTGAACCACGAGAA-AT-3′; 5′-CCTTCCACAATGCCAAAGTT-3′). To control for aberrant splicing or the presence of an internal promoter in the utrophin A 5′UTR, qRT-PCR control experiments were conducted as previously described to detect expression of portions of βGAL and CAT from the bicistronic mRNA transcript [16], [43], [56]. For these experiments, the TRIzol extracted RNA was treated for 1 hour with DNAse I (Invitrogen) to prevent DNA contamination from the transfected plasmid. Reverse transcription for all qRT-PCR experiments was carried out using the First-Strand cDNA Synthesis kit (Amersham Biosciences, Piscataway, NJ) with NotI-d(T)18 primers. Quantitative real time PCR was performed using the QuantiTect SYBR green PCR kit (Qiagen, Mississauga, ON, Canada). Analysis was performed using the ABI Prism 7000 sequence detection system (Applied Biosystems, Foster City, CA) with ABI Prism 7000 SDS Software. To control for DNA contamination, quantitative PCR was performed directly on the RNA samples.

### Statistical Analysis

The presence of significant differences between group means was determined using Student's t test. The level of significance was set at p<0.05. Means +/− S.E. are shown throughout.
